# Supplementary figures and images for: DeNovoCNN: a deep learning approach to de novo variant calling in next generation sequencing data
Source: Nucleic Acids Res. 2022 Jun 17;50(17):e97. doi: 10.1093/nar/gkac511 (PMC9508836; doi:10.1093/nar/gkac511)

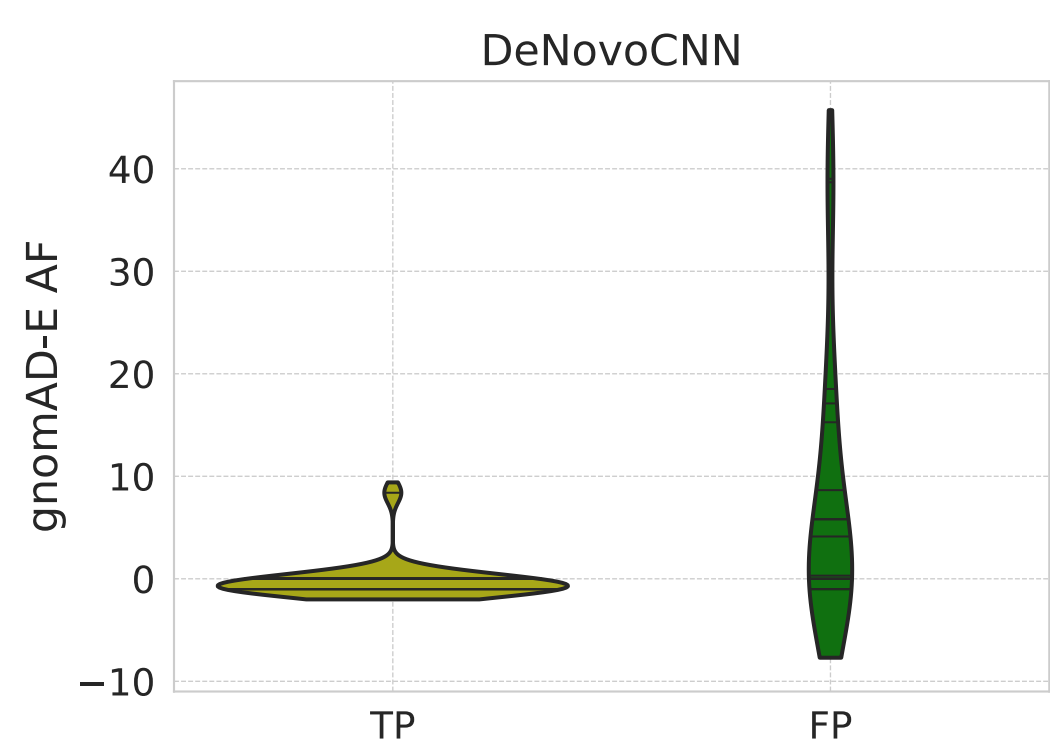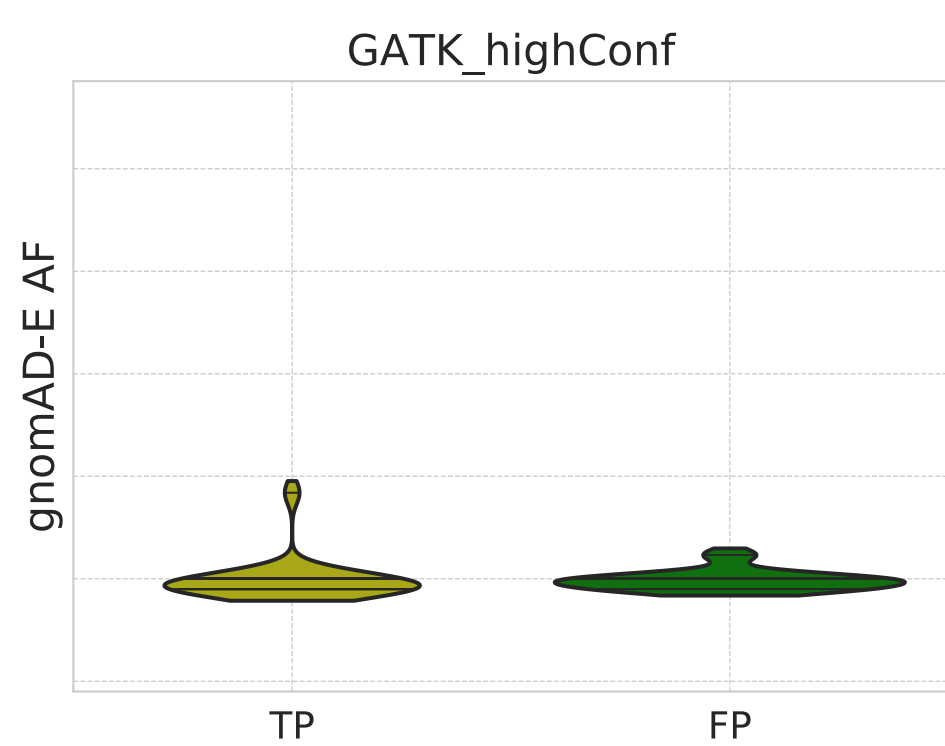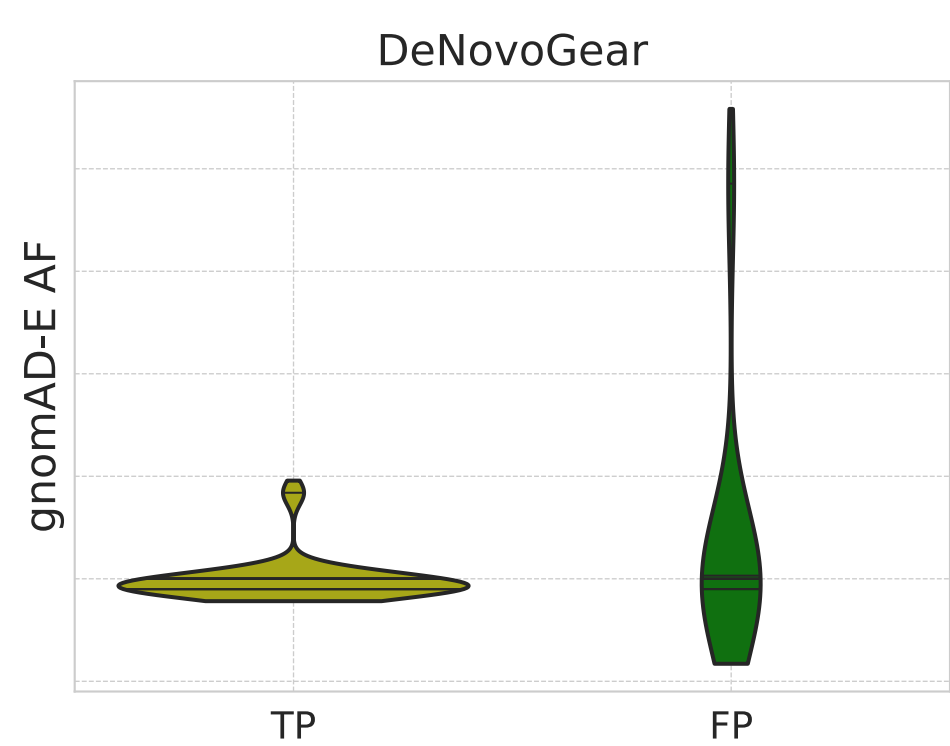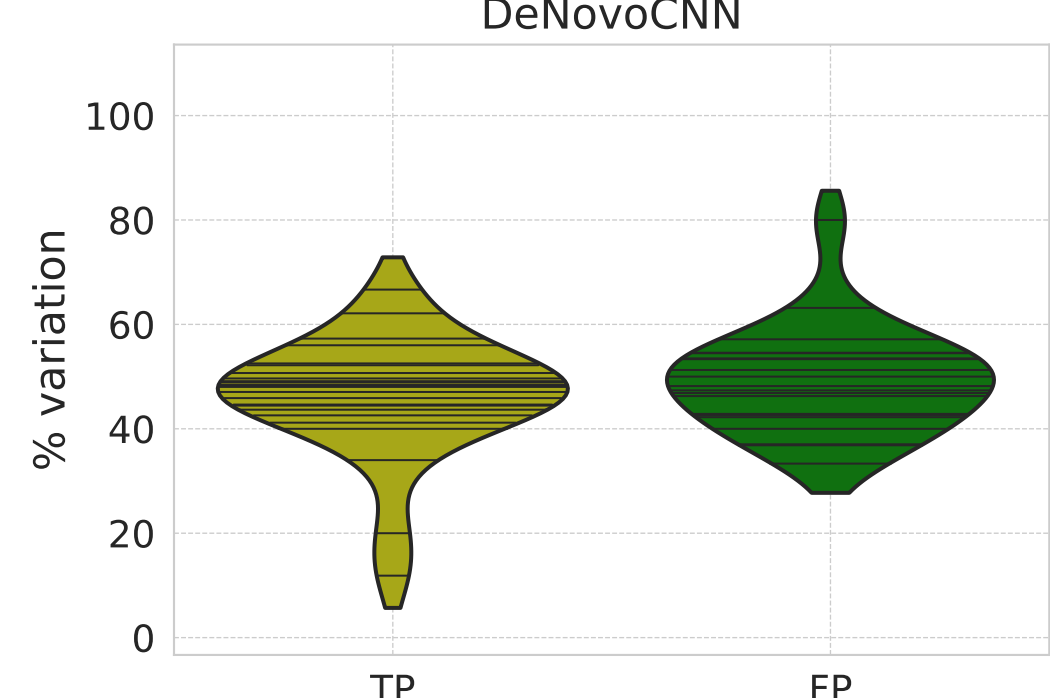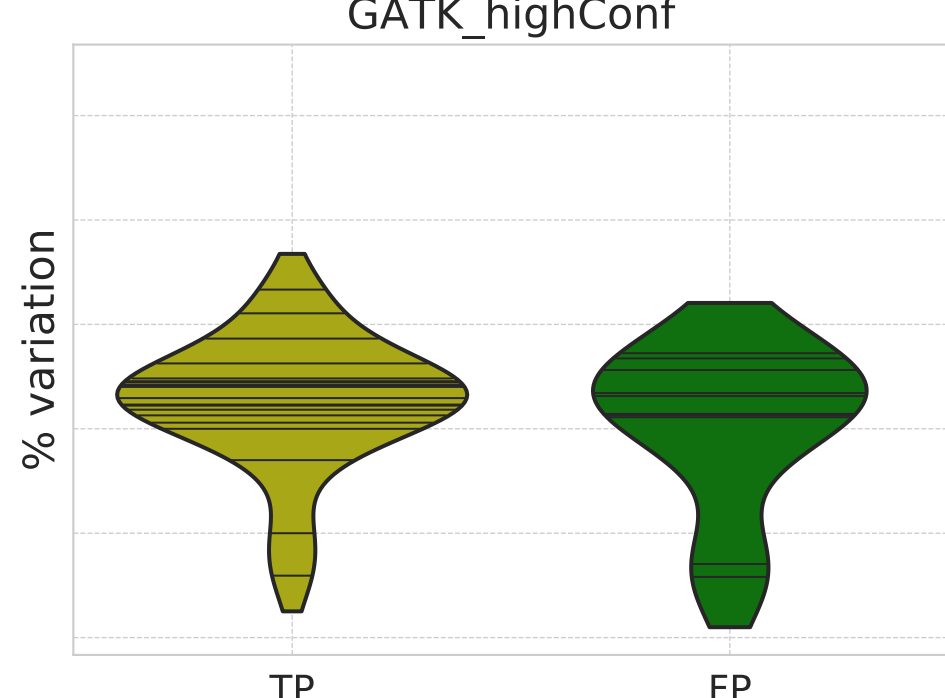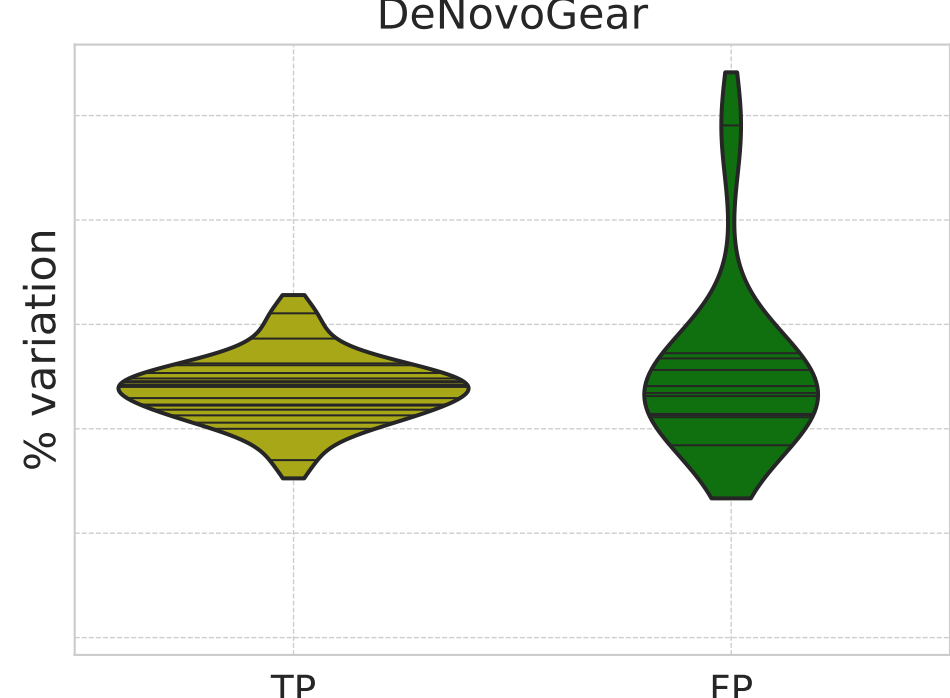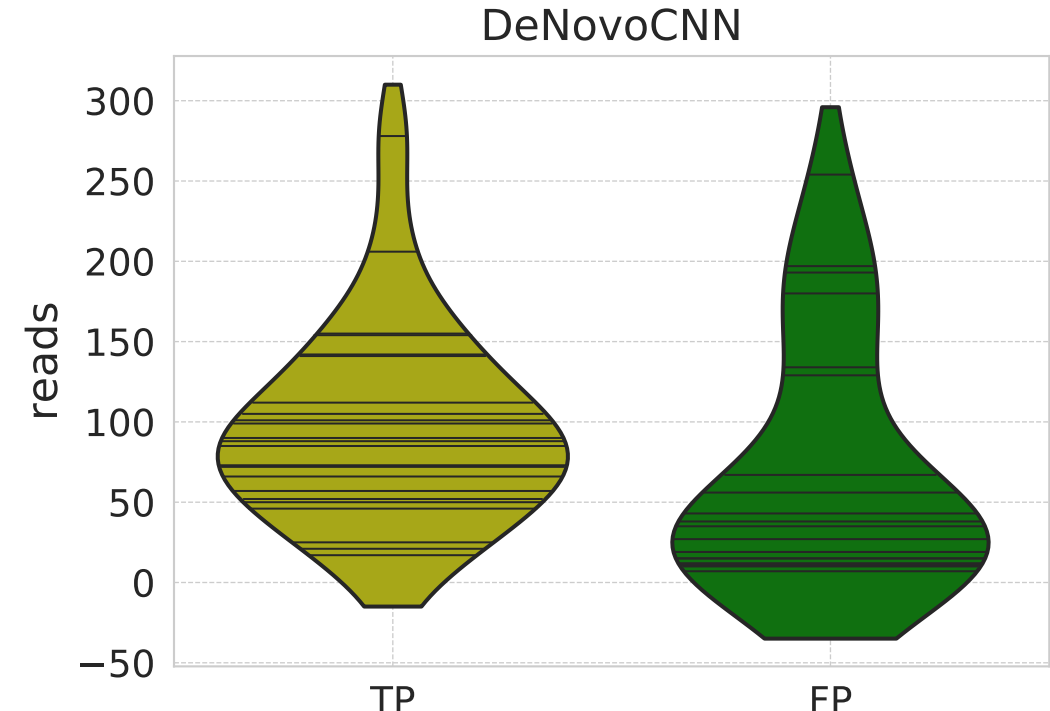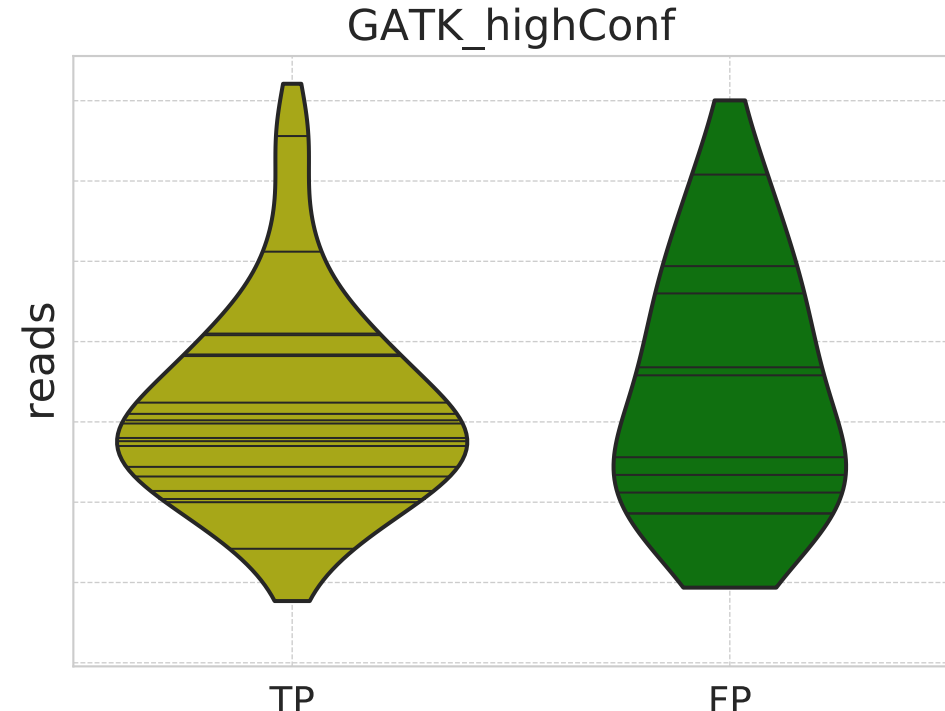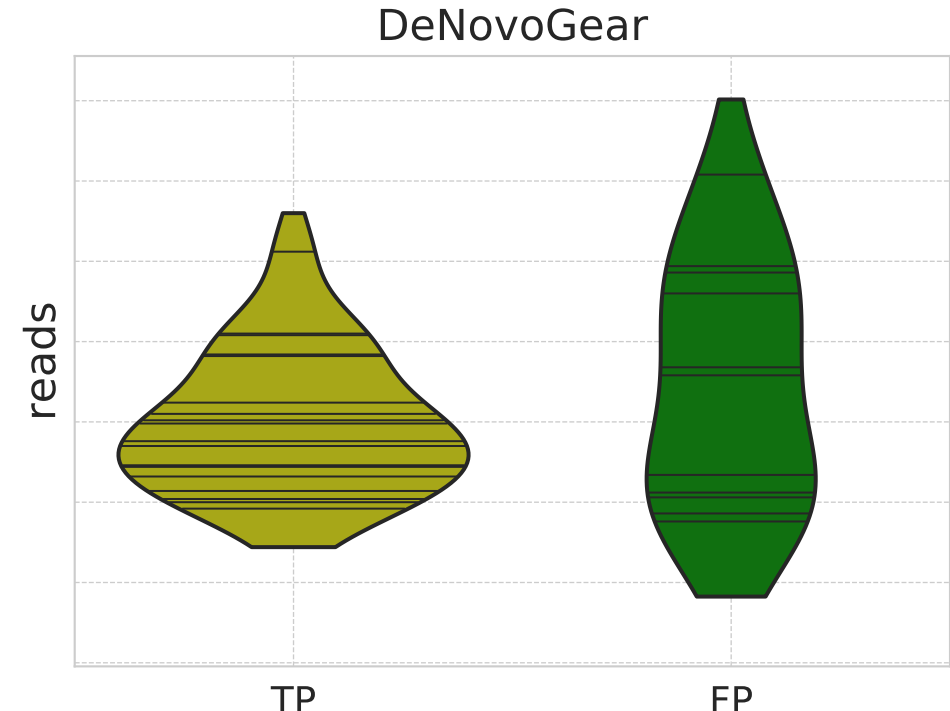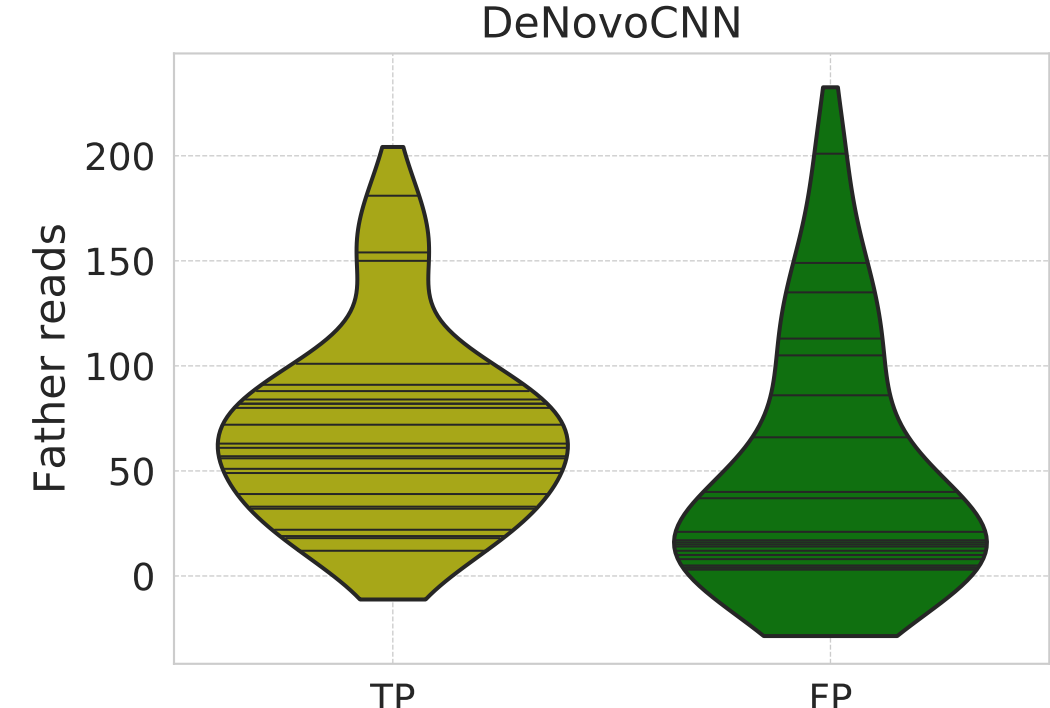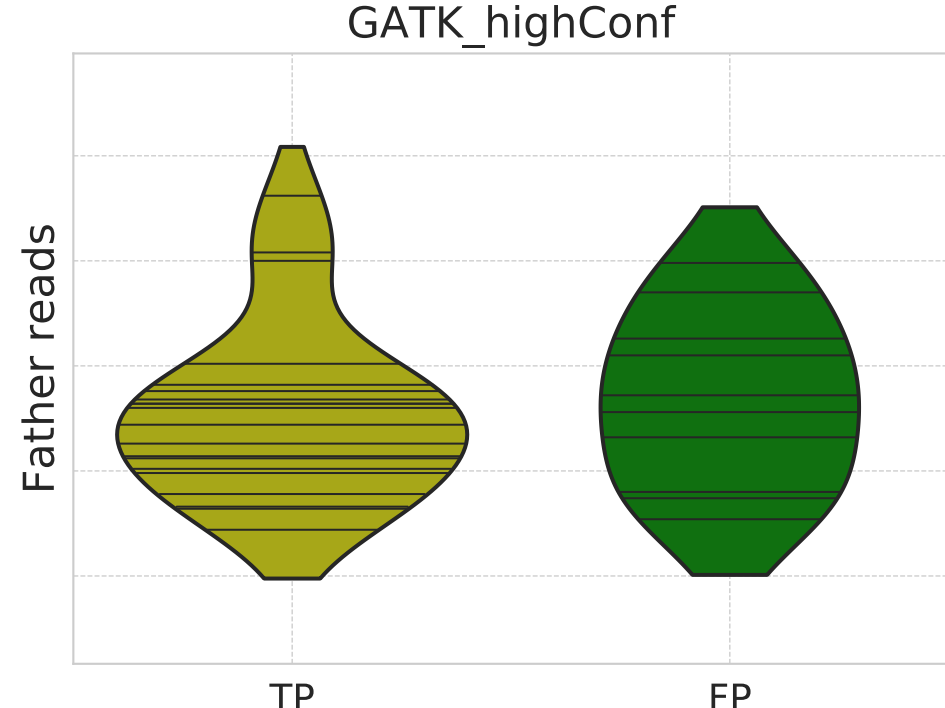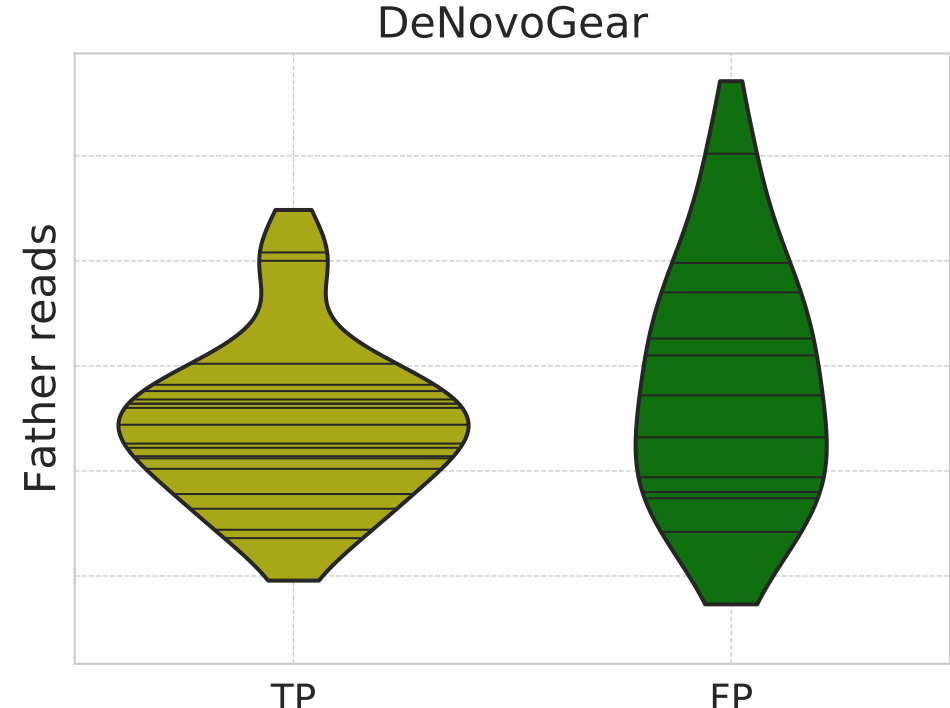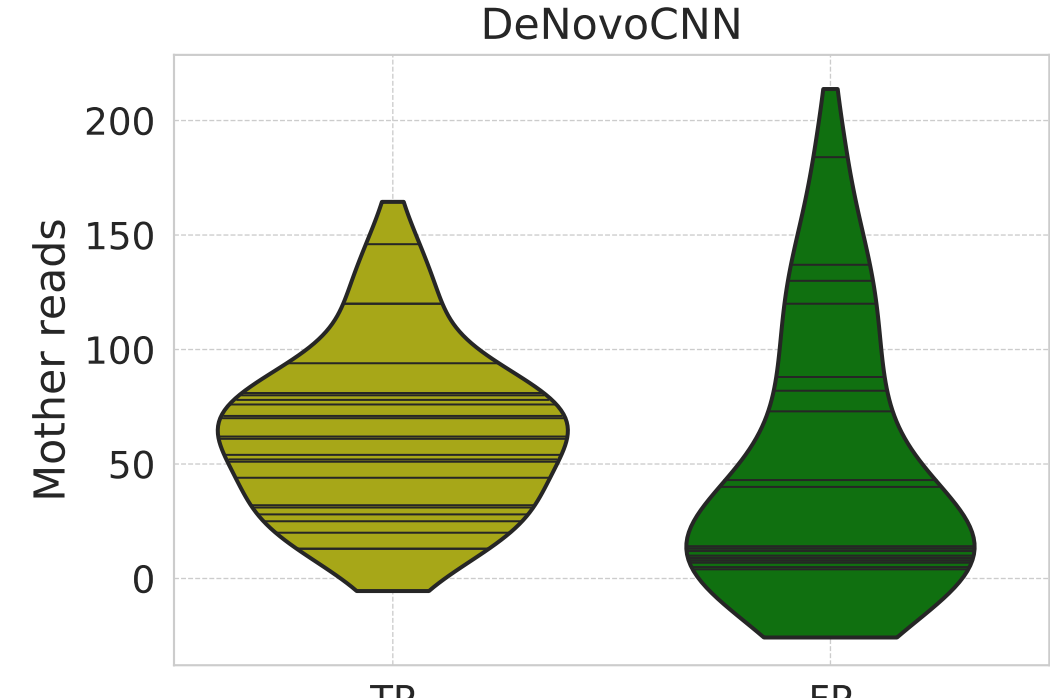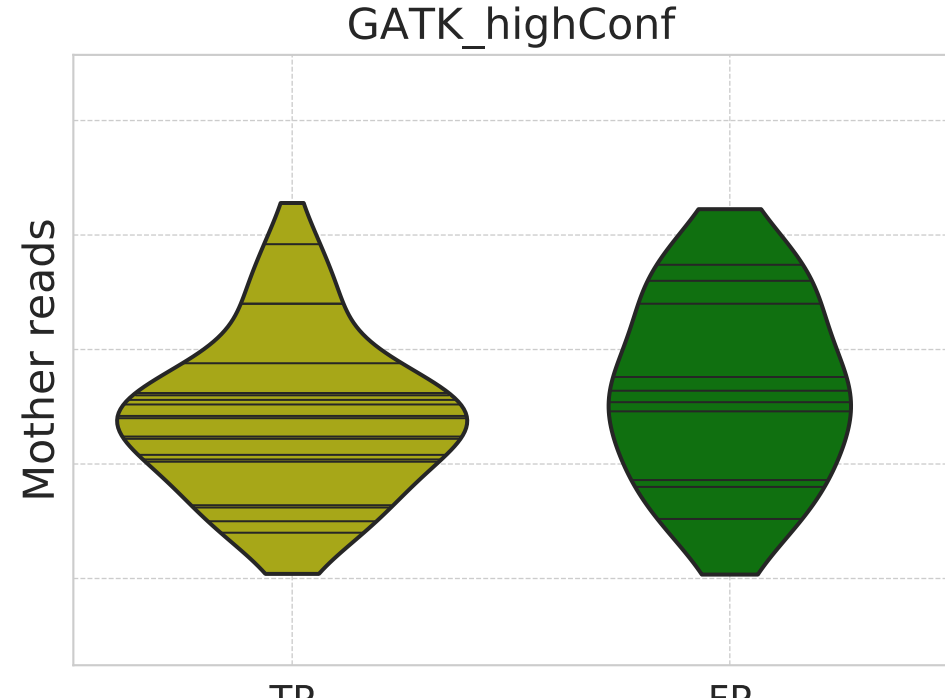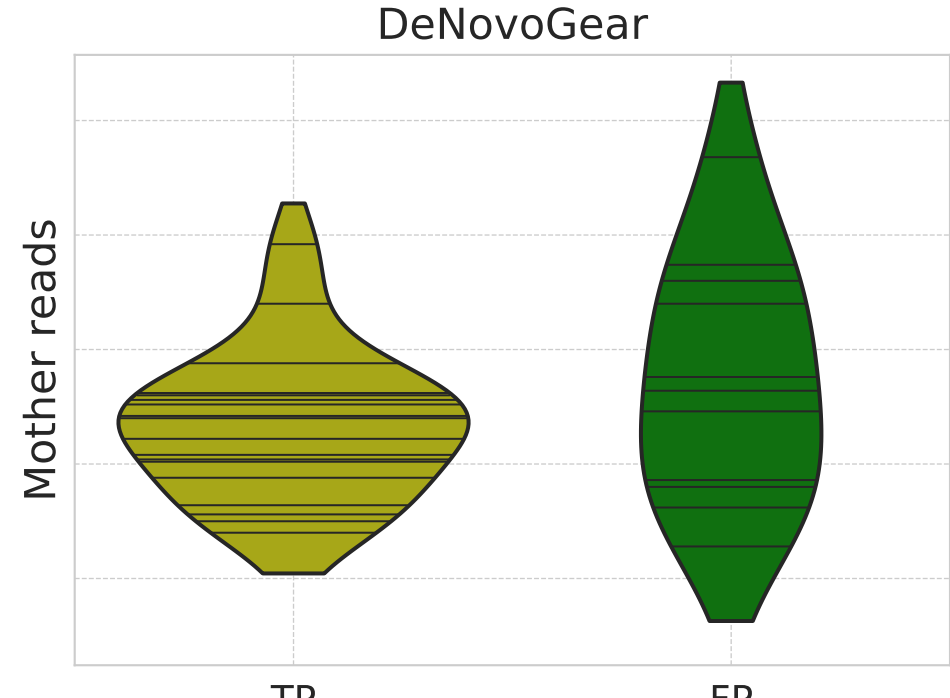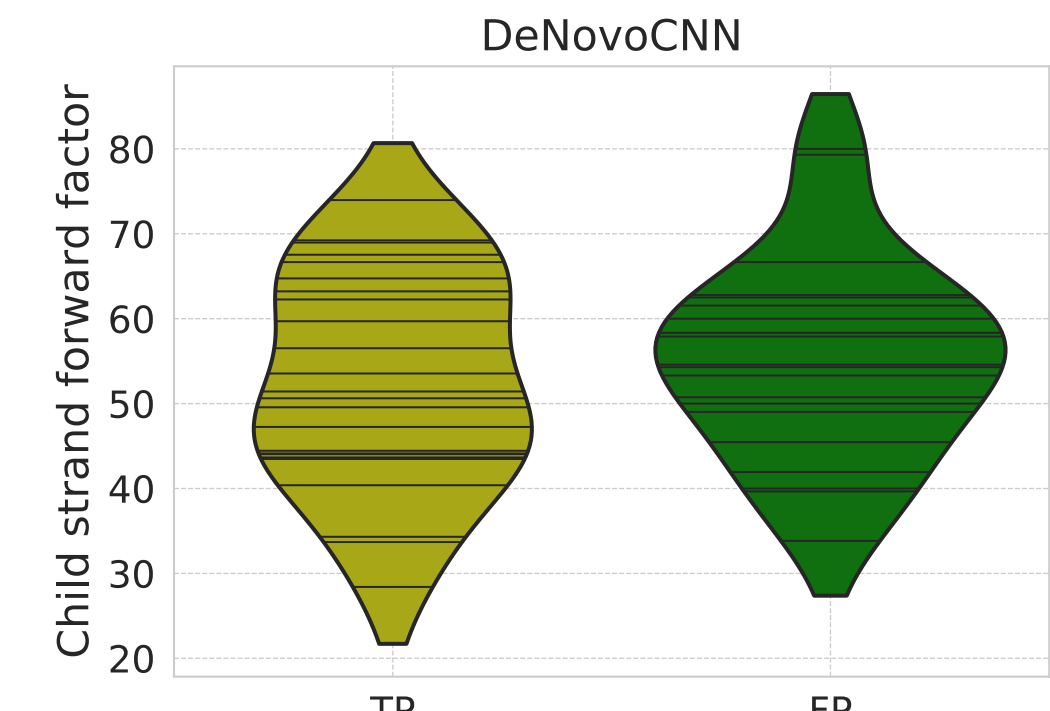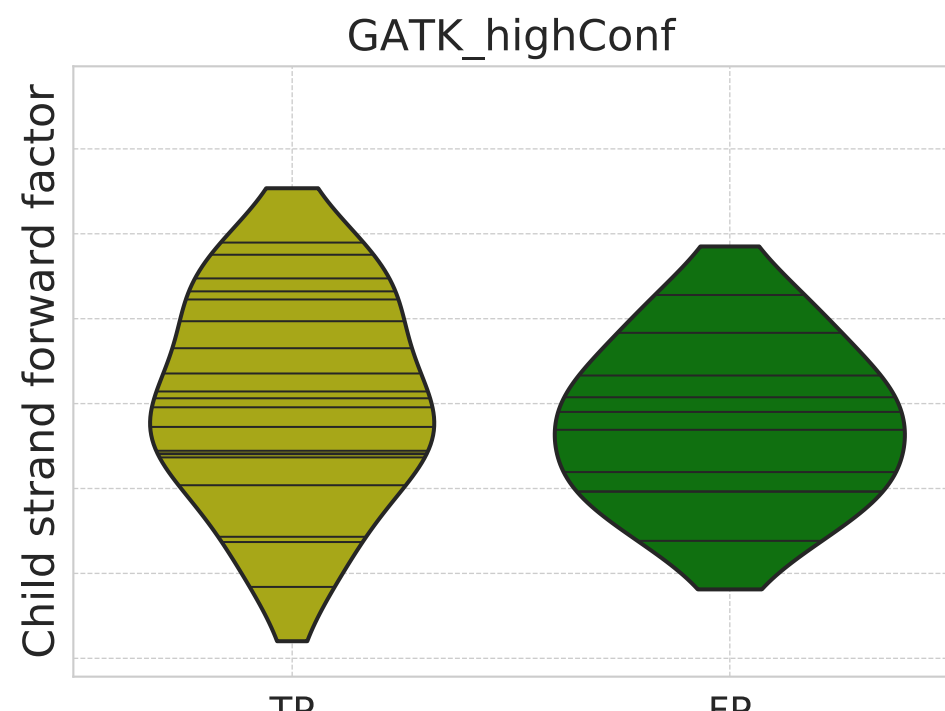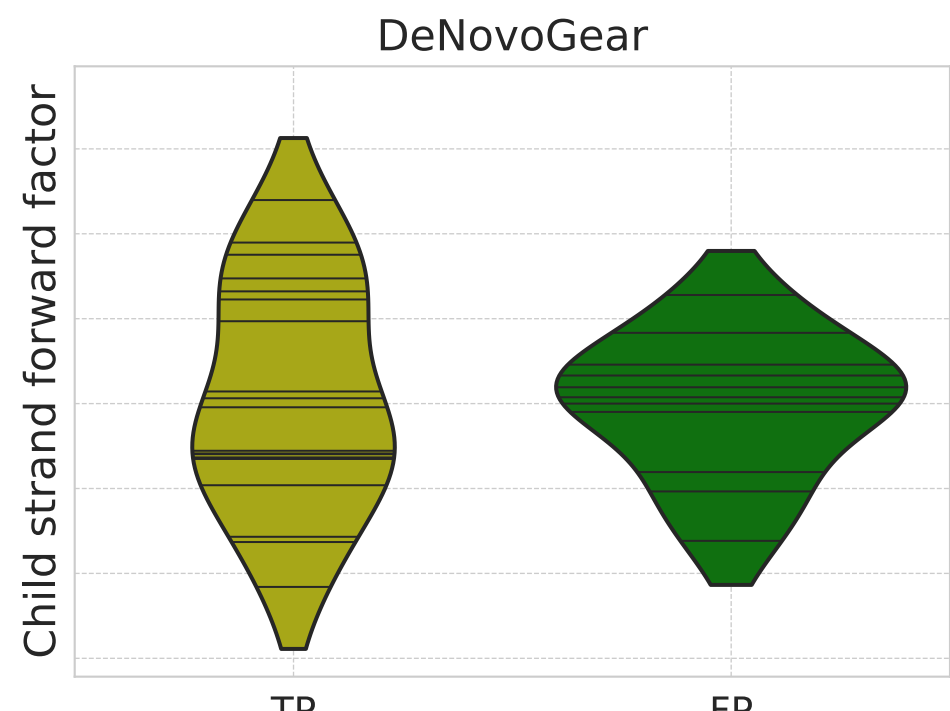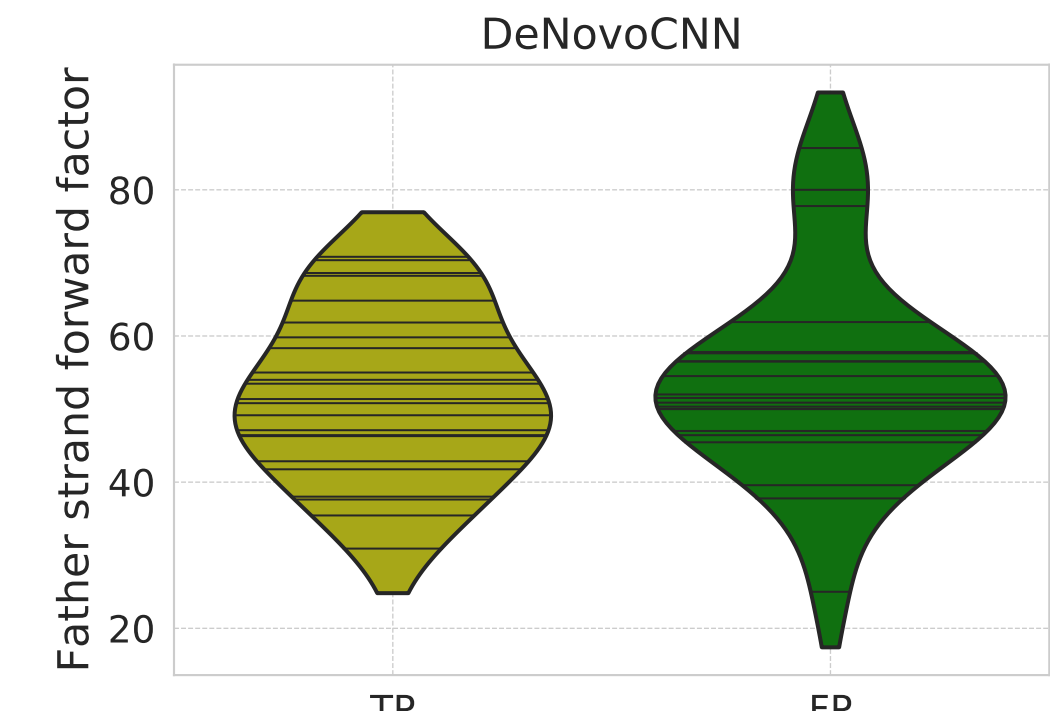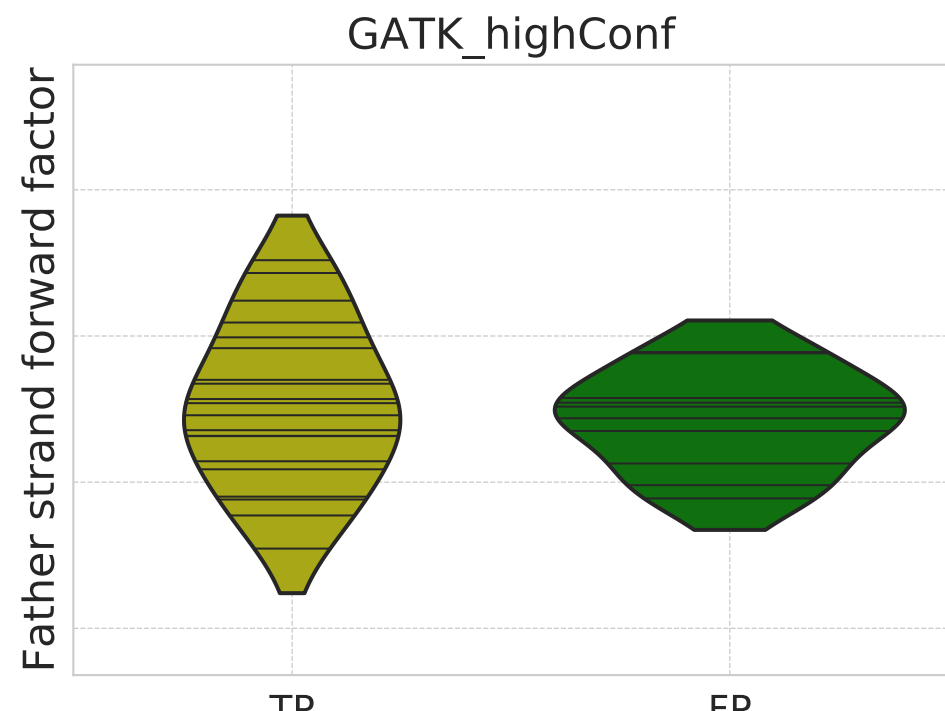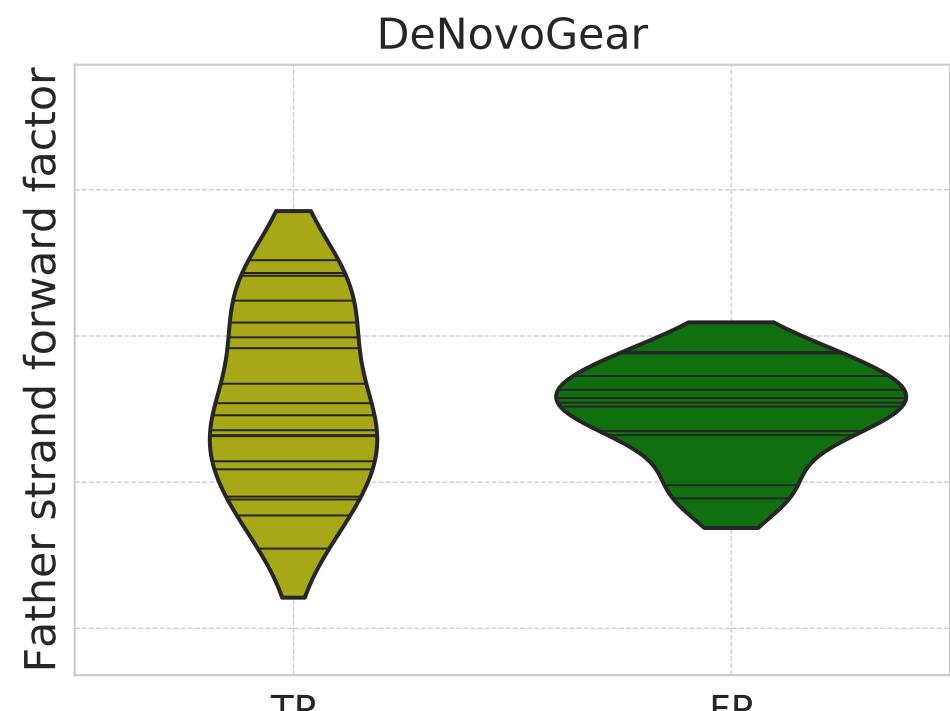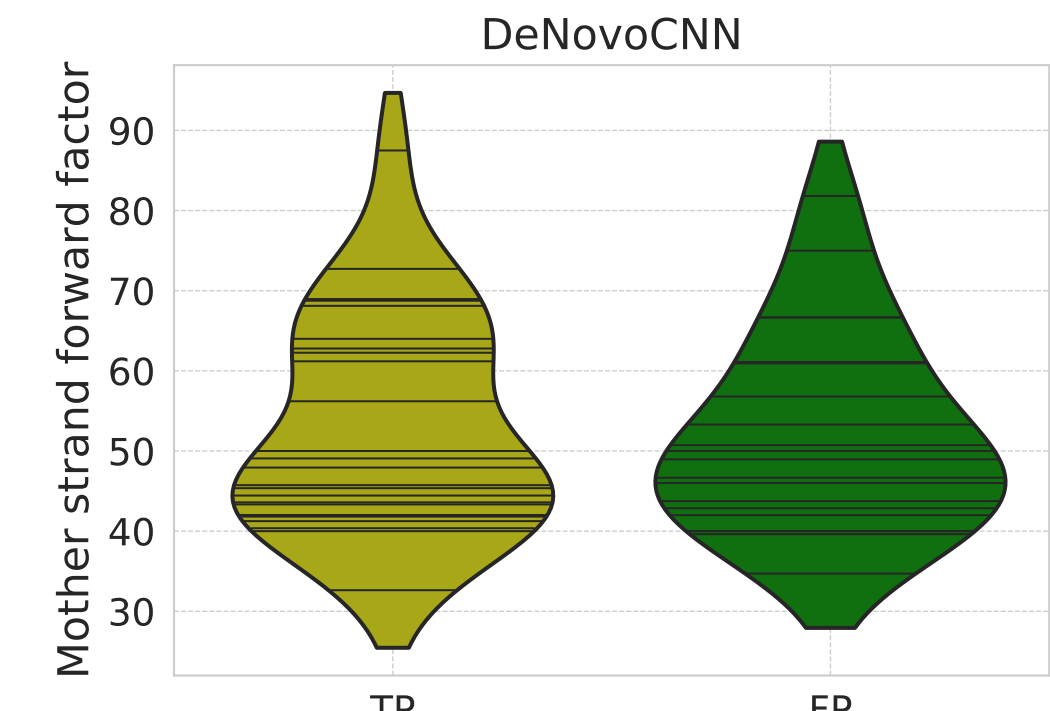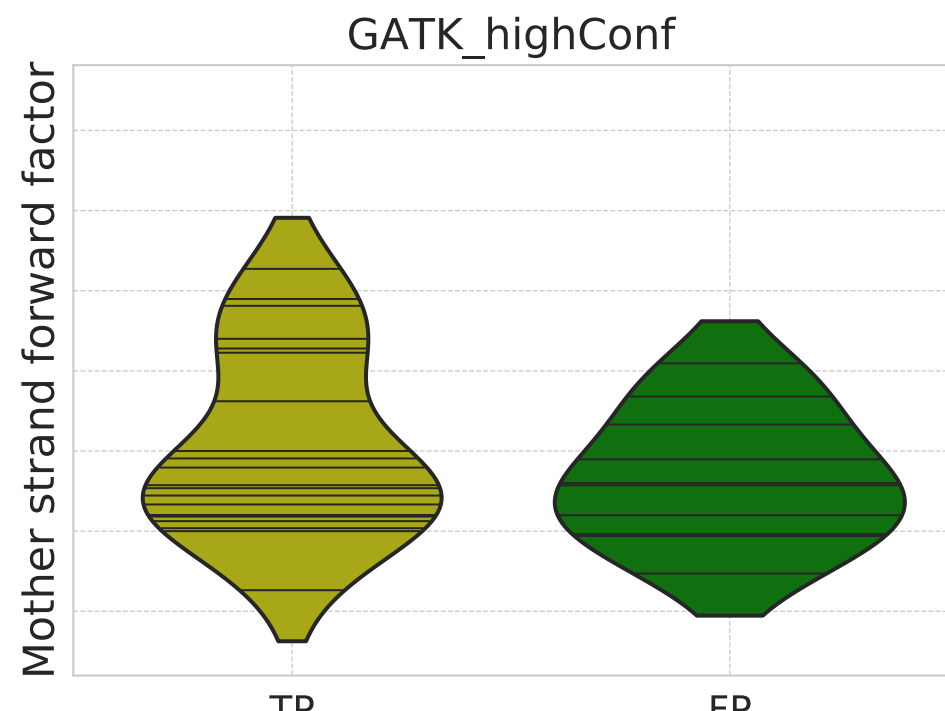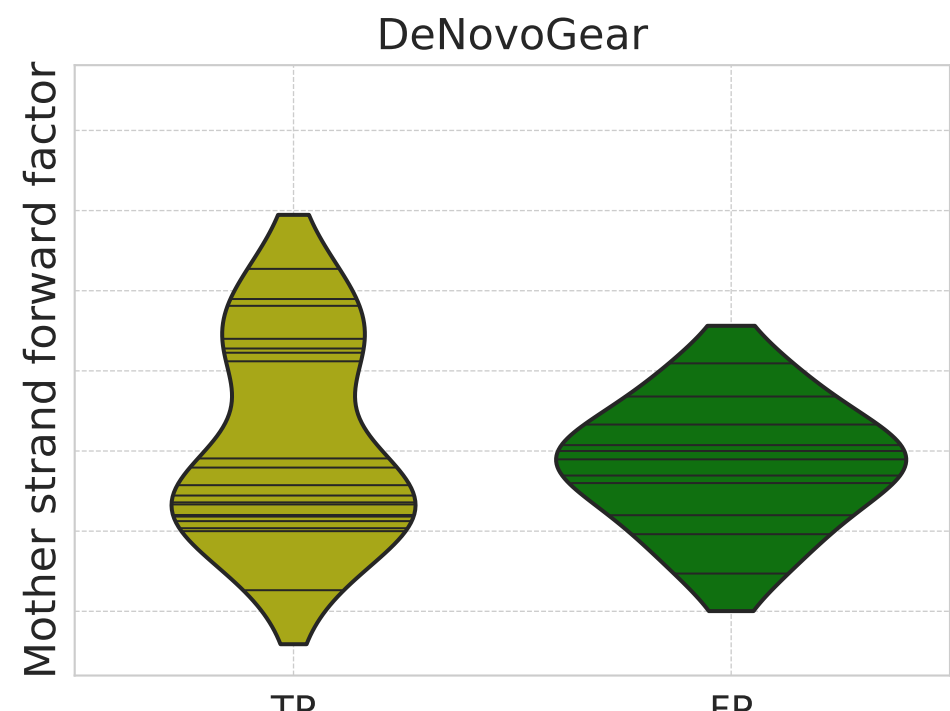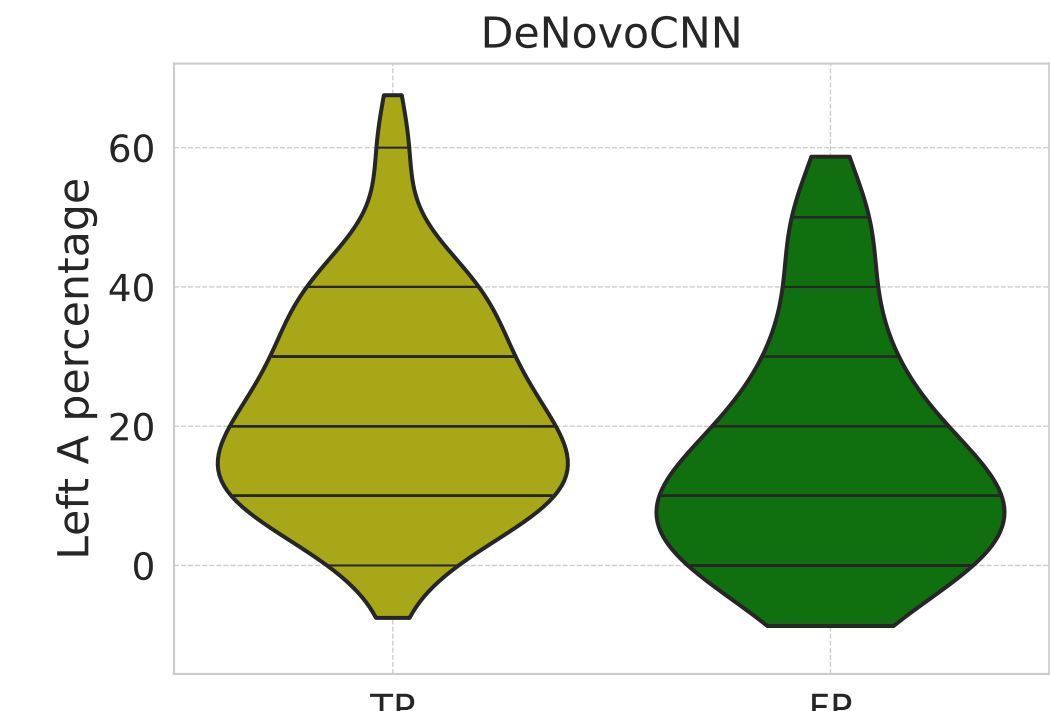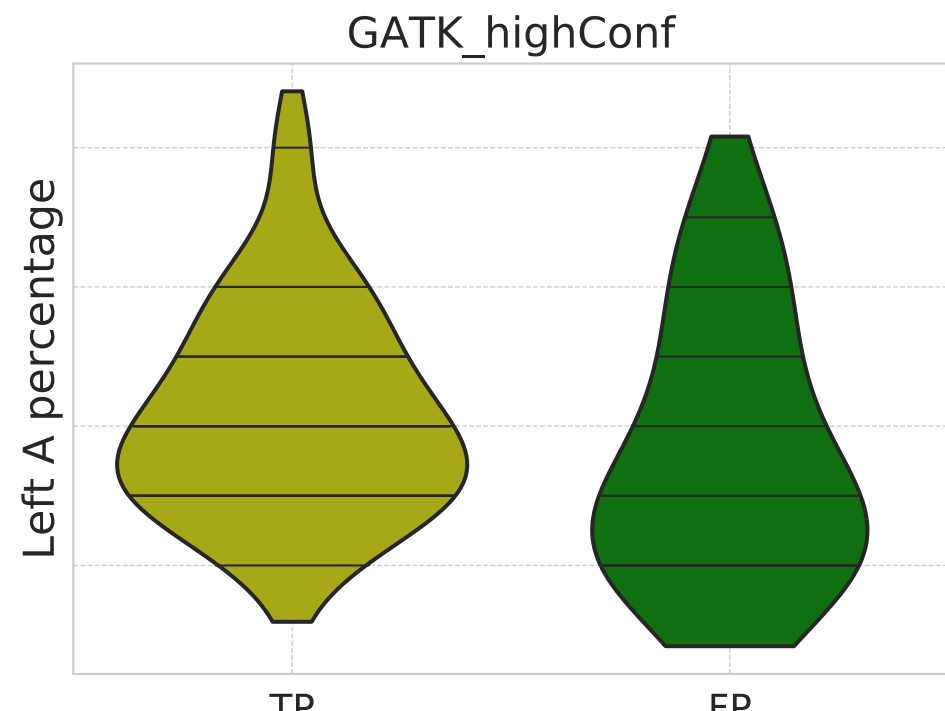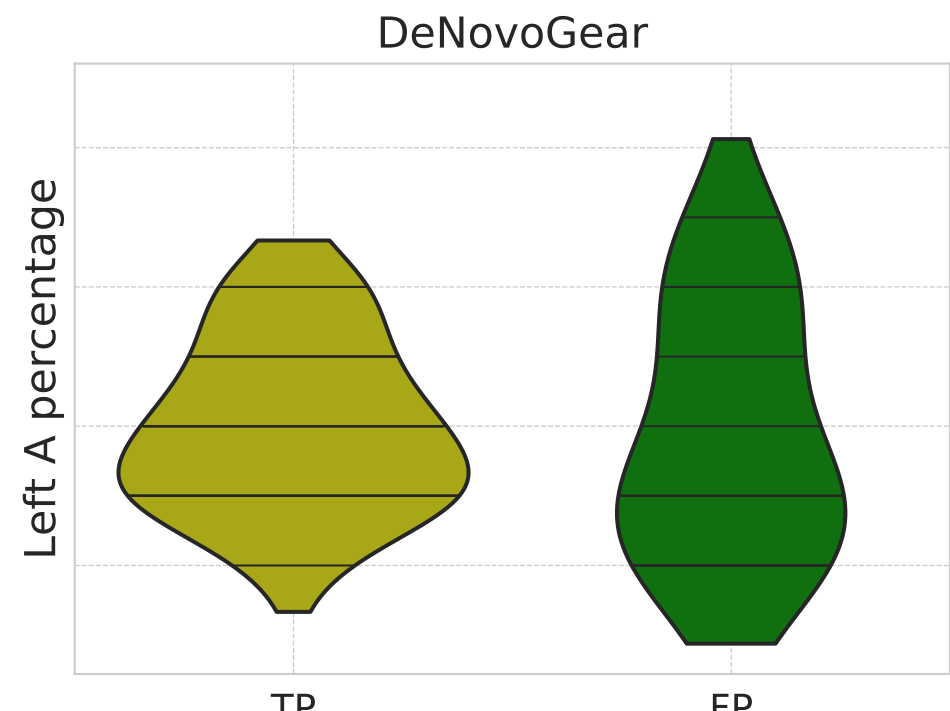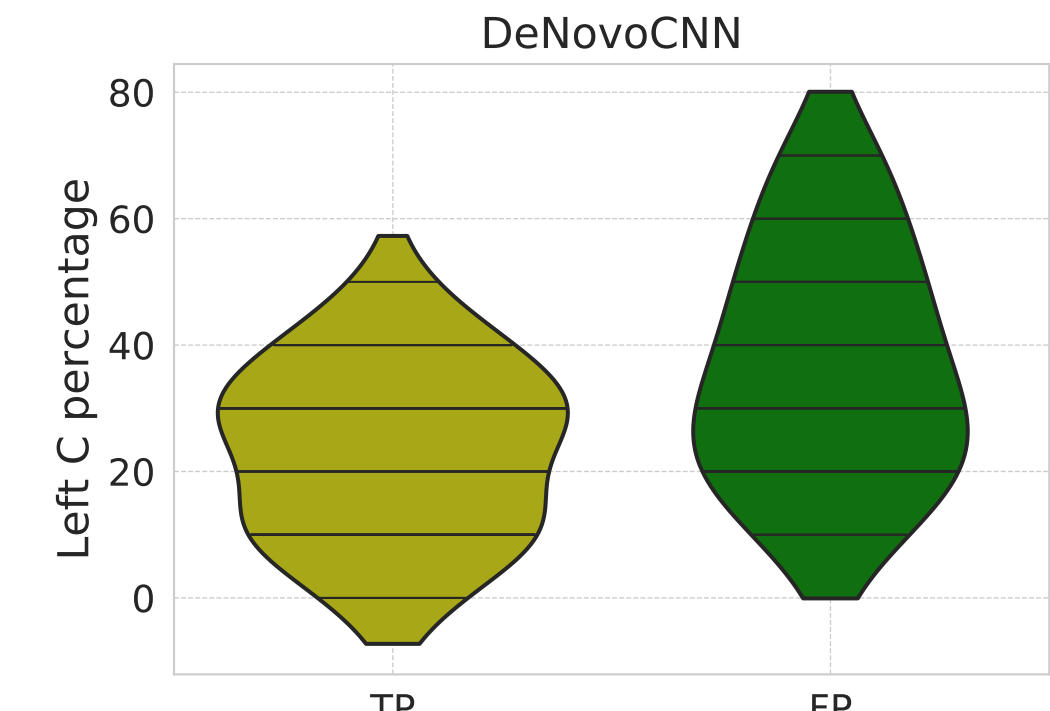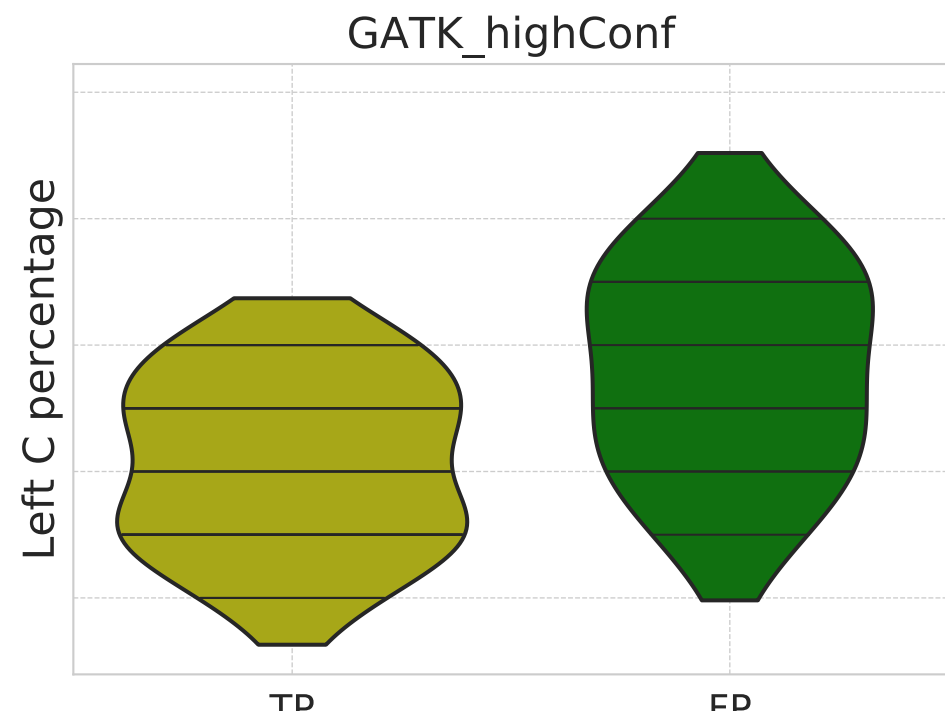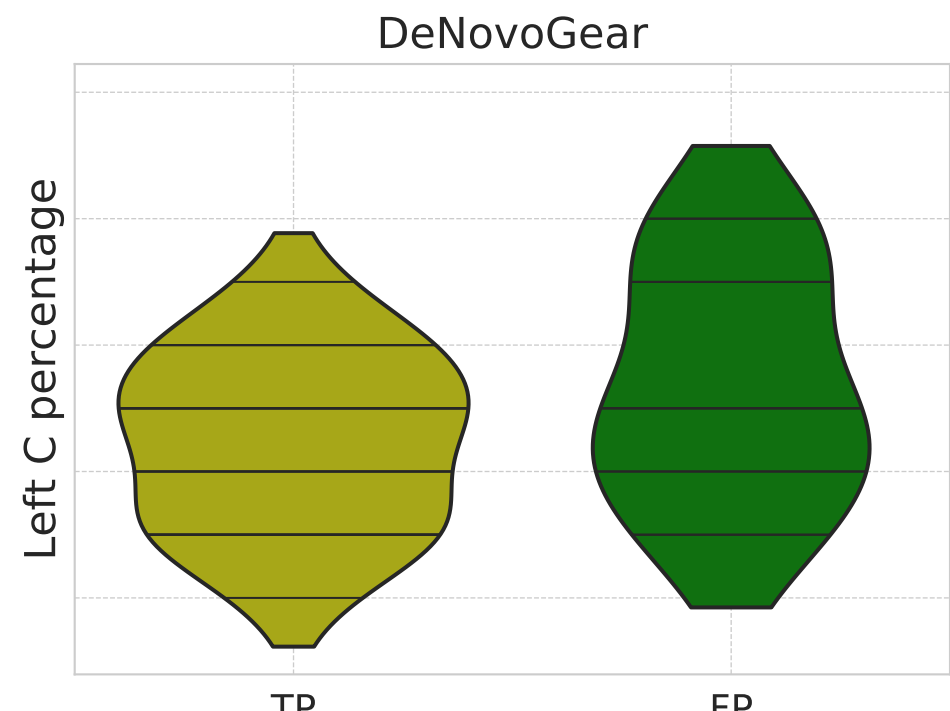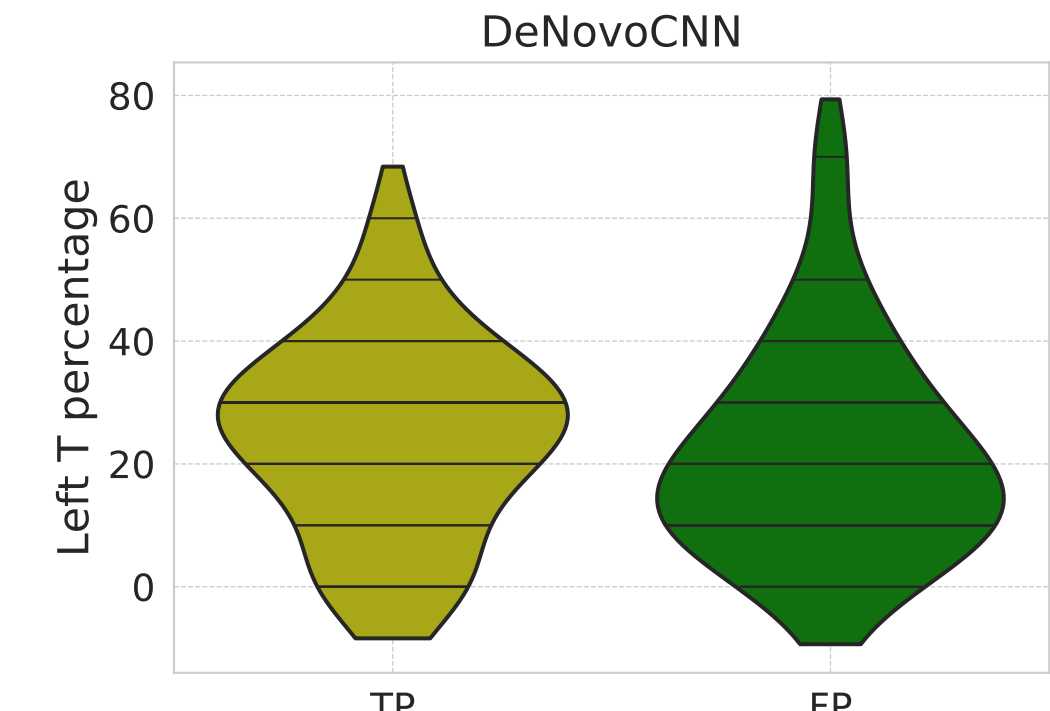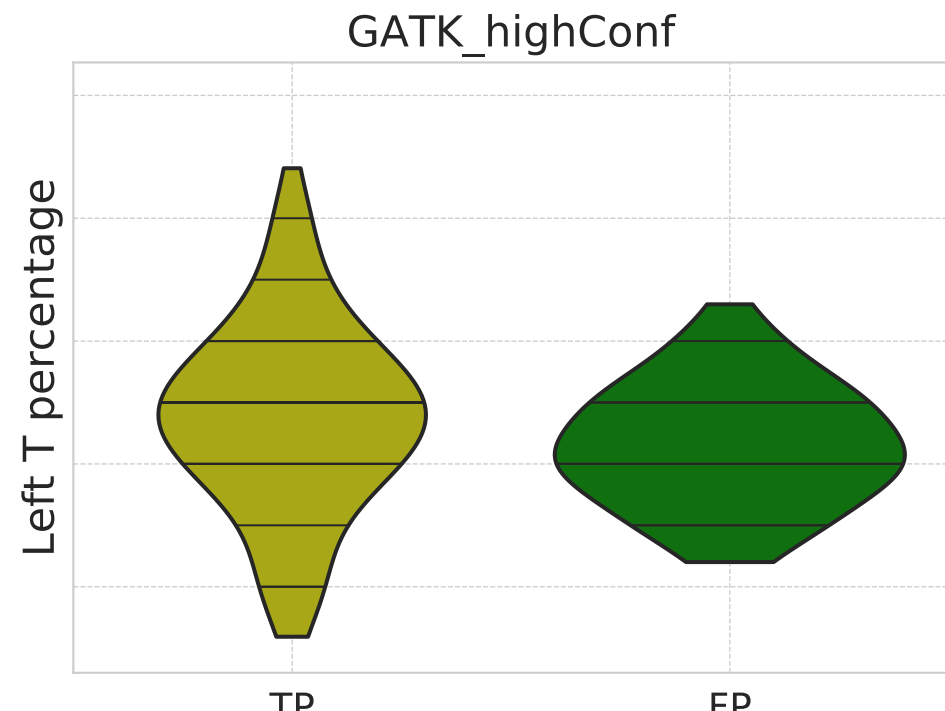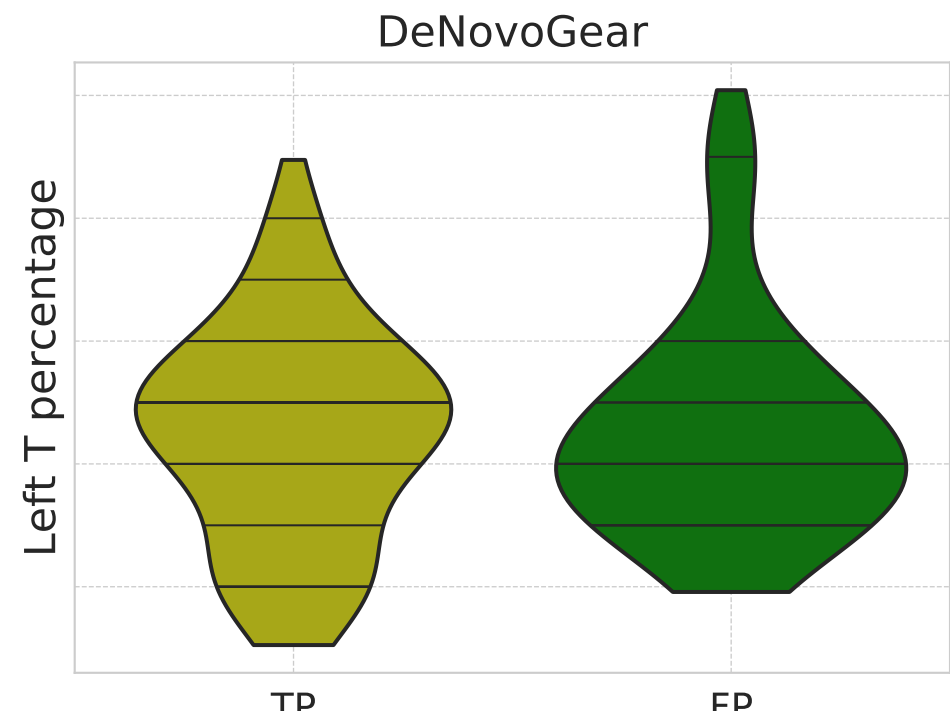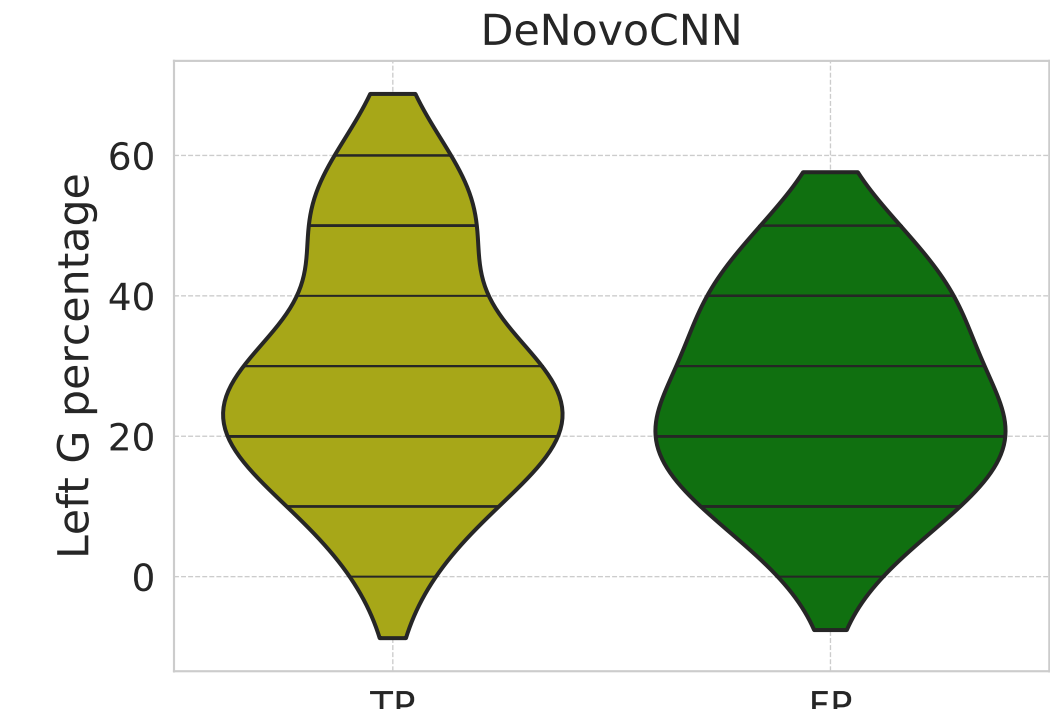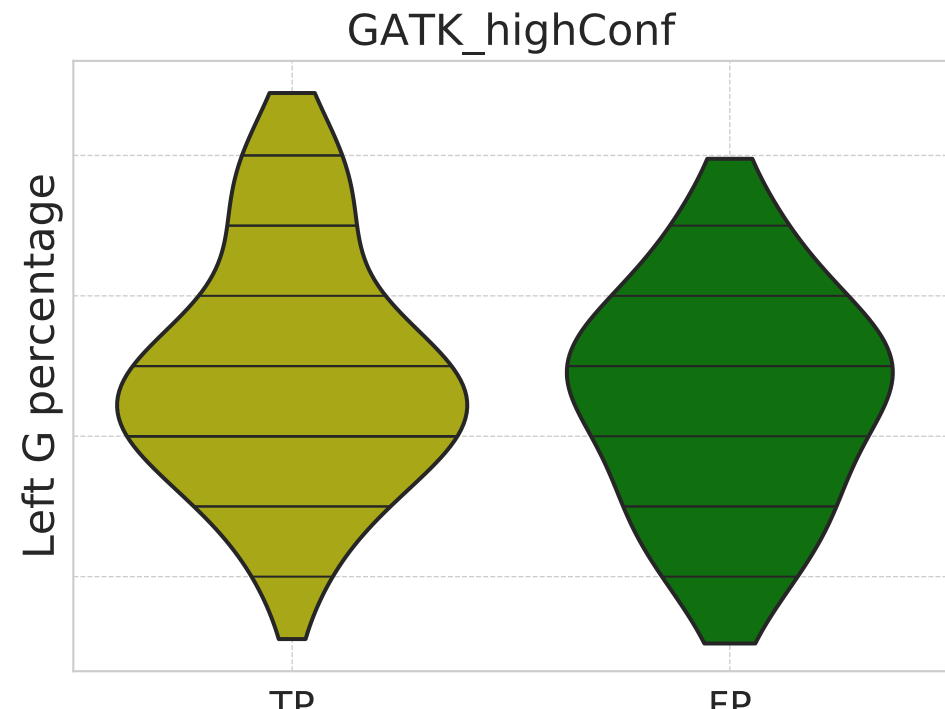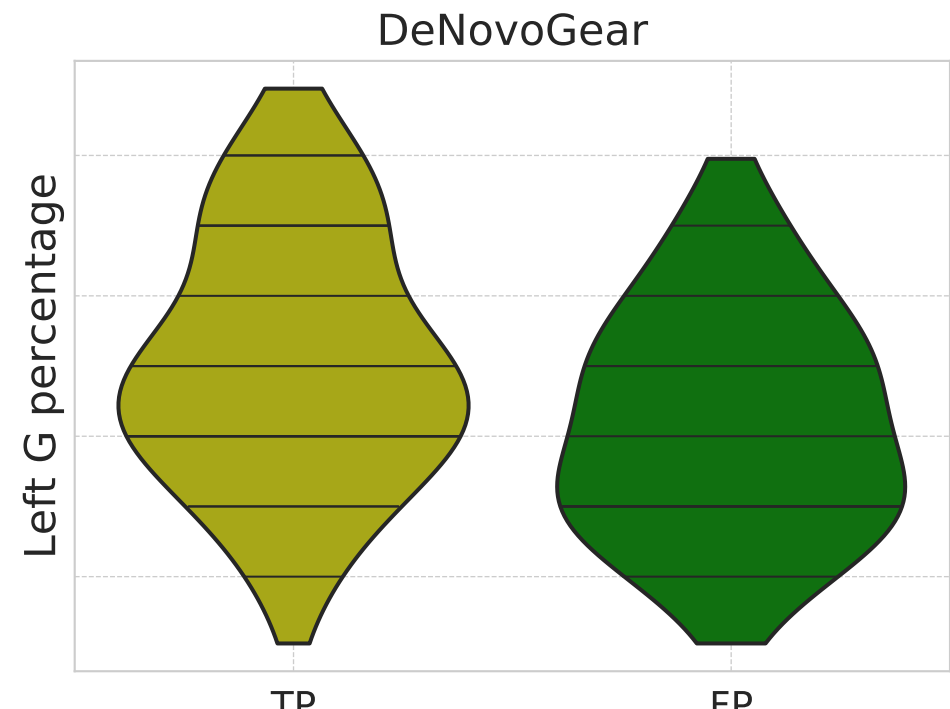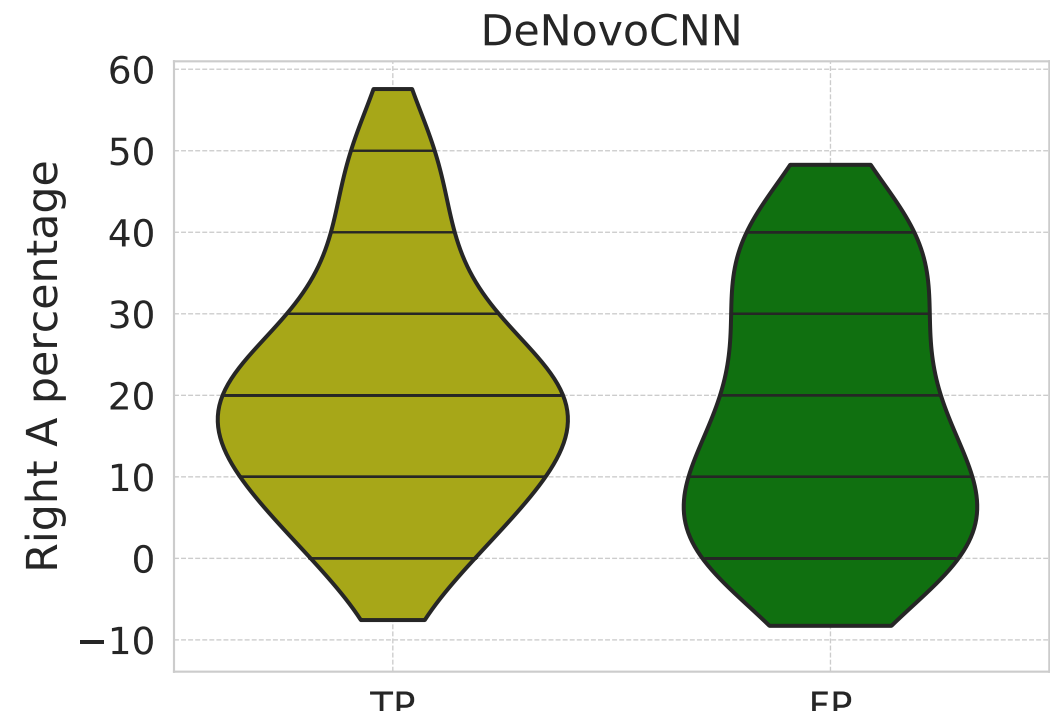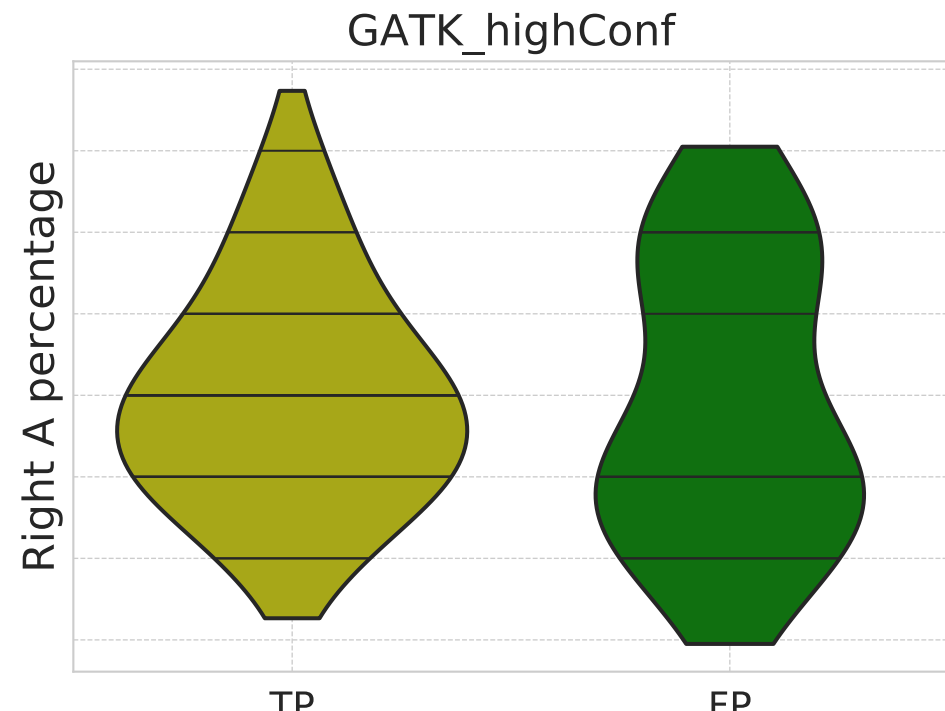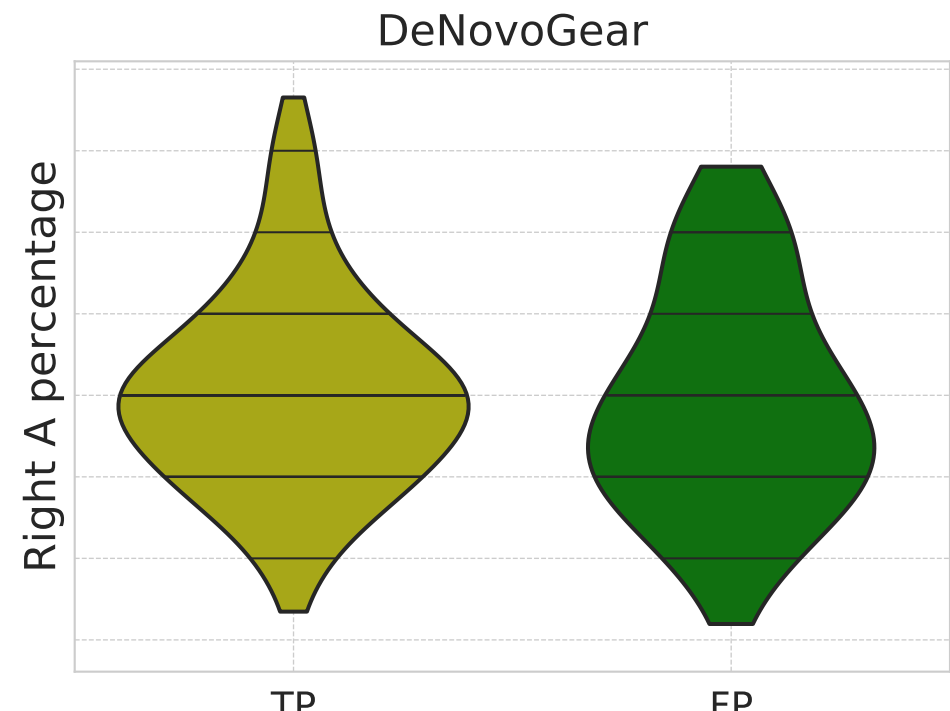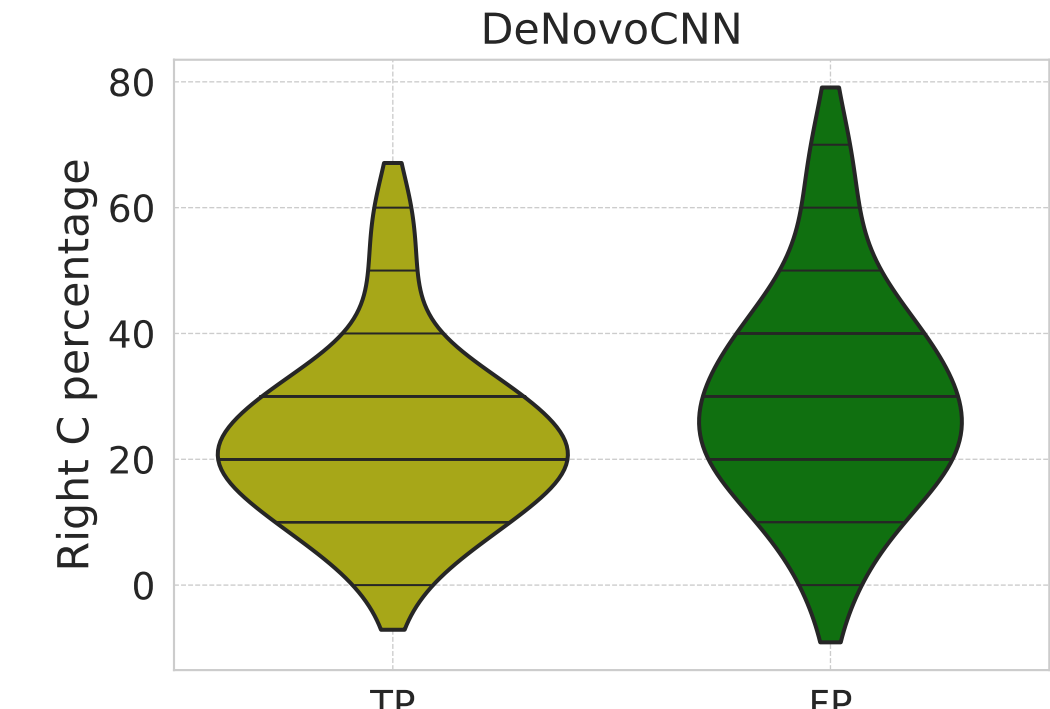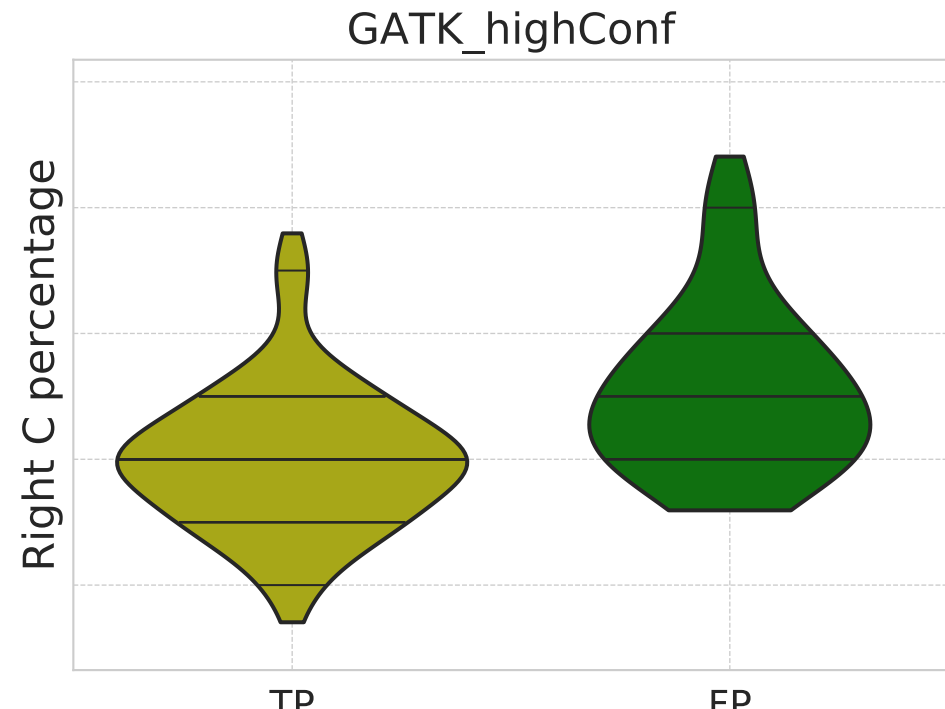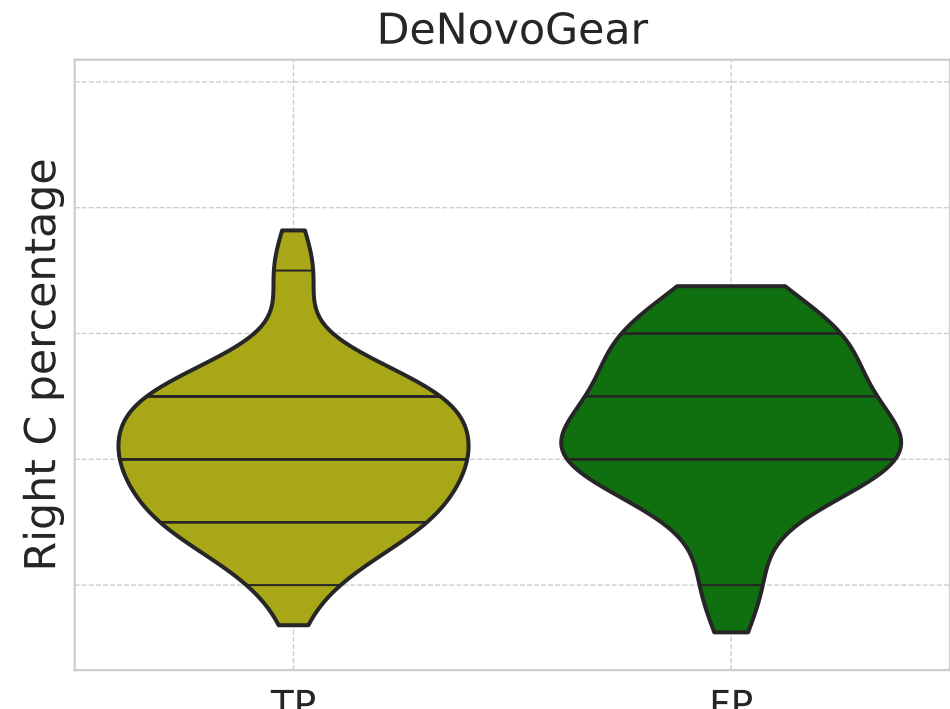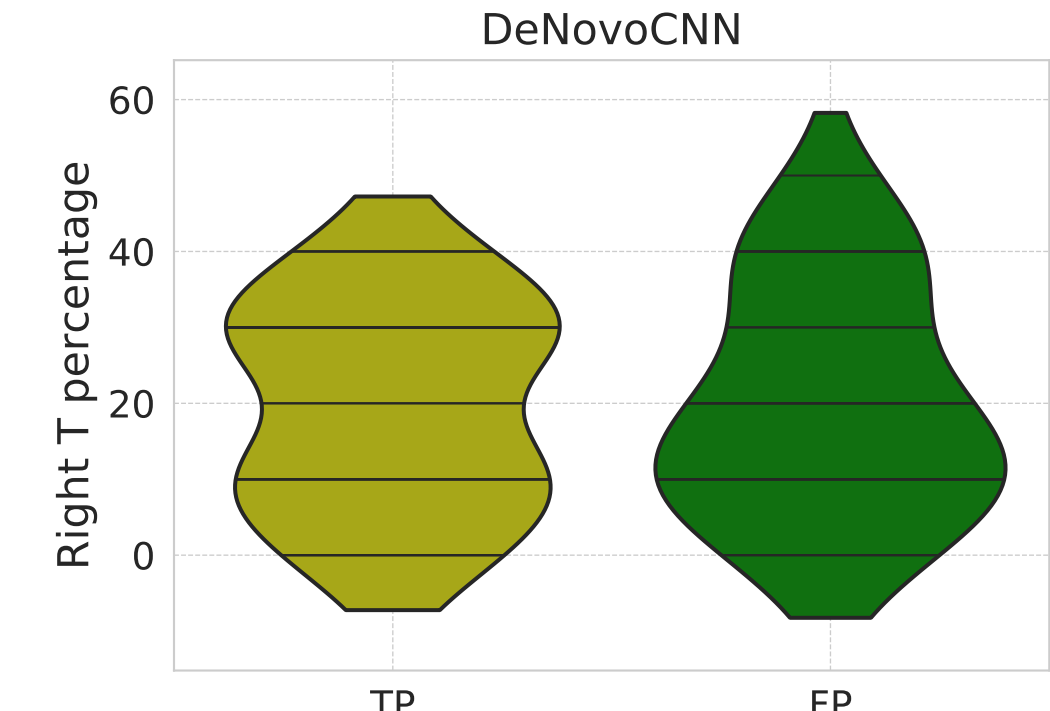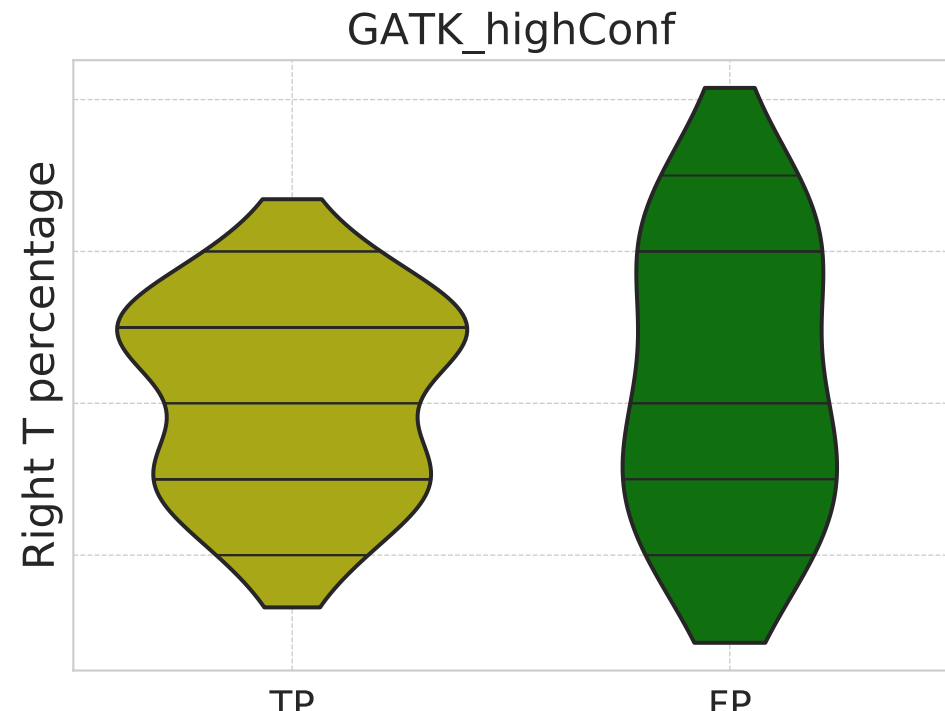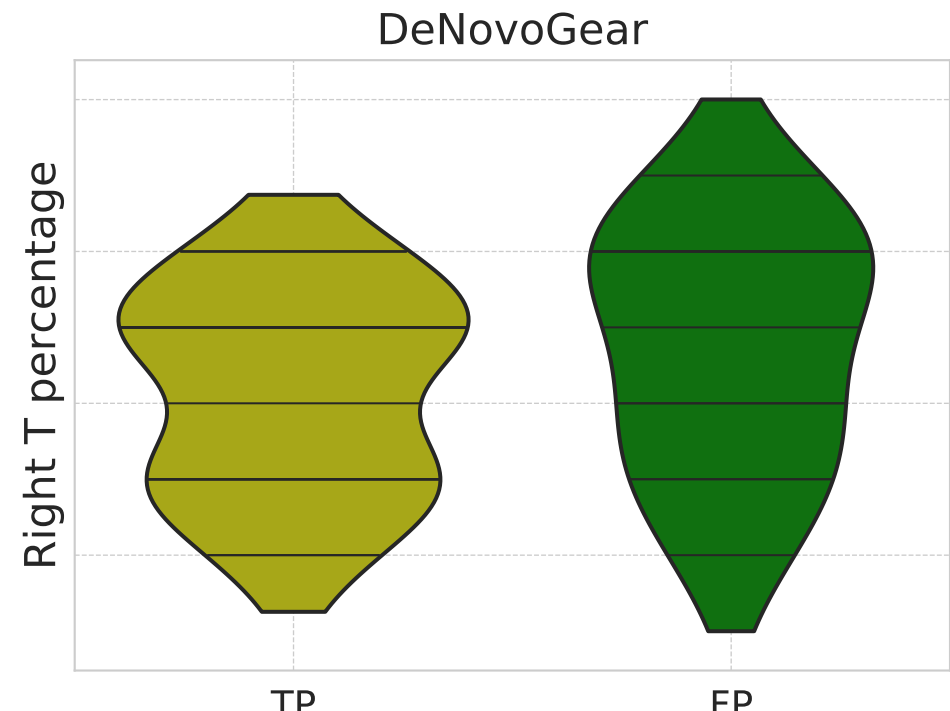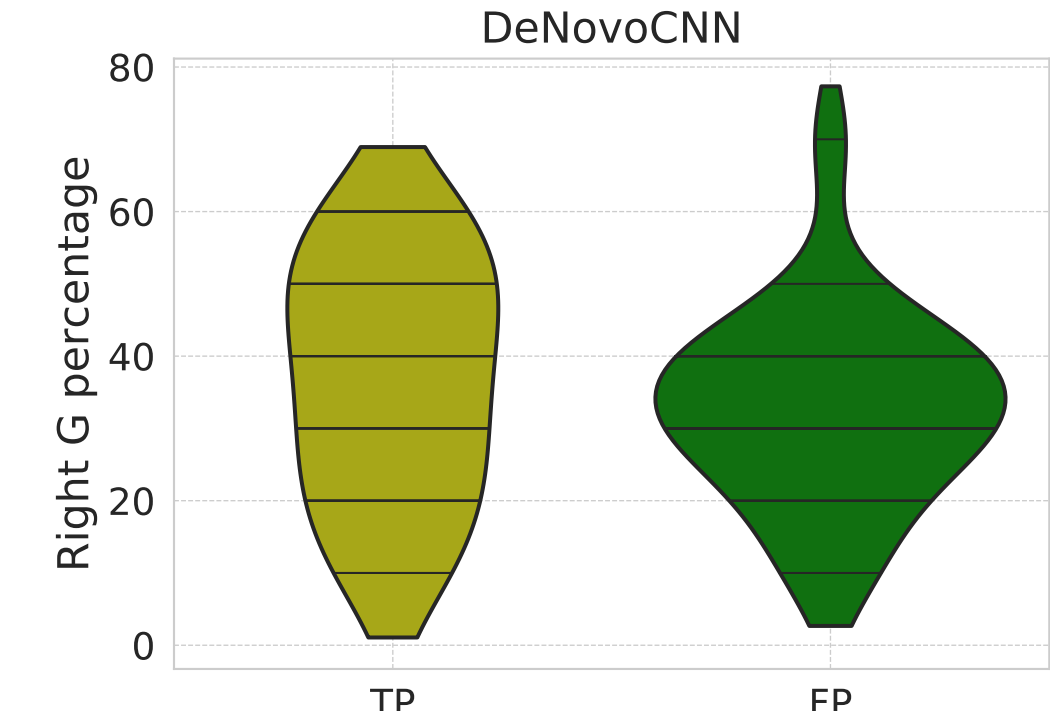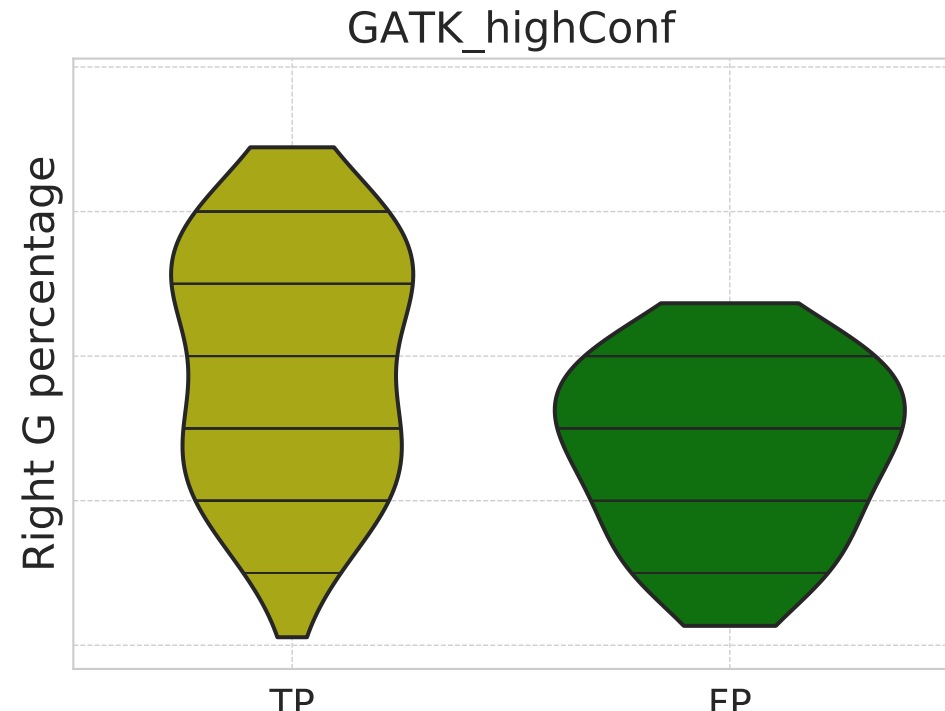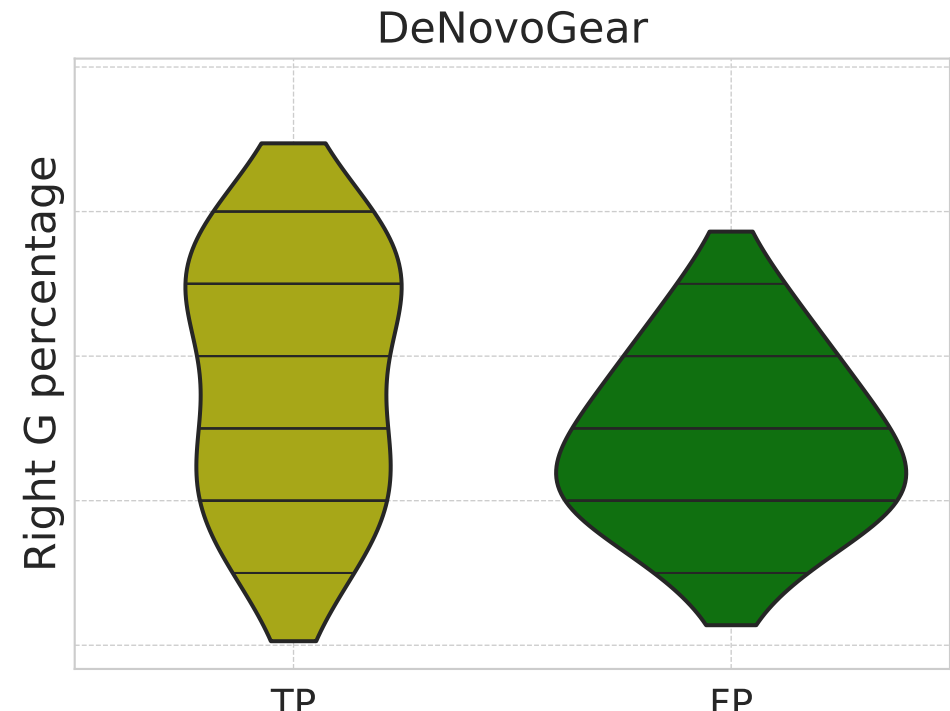

Supplement: gkac511_Supplemental_Files [file gkac511_supplemental_files.zip › 20_wes_FP_comparison.pdf]
